# Genetic Etiology Shared by Multiple Sclerosis and Ischemic Stroke

**DOI:** 10.3389/fgene.2020.00646

**Published:** 2020-07-03

**Authors:** Zhu Tian, Yang Song, Yang Yao, Jie Guo, Zhongying Gong, Zhiyun Wang

**Affiliations:** Department of Neurology, Tianjin First Central Hospital, Tianjin, China

**Keywords:** multiple sclerosis, ischemic stroke, genome-wide association studies, gene-based test, gene expression analyses

## Abstract

Although dramatic progress has been achieved in the understanding and treatment of multiple sclerosis (MS) and ischemic stroke (IS), more precise and instructive support is required for further research. Recent large-scale genome-wide association studies (GWASs) have already revealed risk variants for IS and MS, but the common genetic etiology between MS and IS remains an unresolved issue. This research was designed to overlapping genes between MS and IS and unmask their transcriptional features. We designed a three-section analysis process. Firstly, we computed gene-based analyses of MS GWAS and IS GWAS data sets by VGEAS2. Secondly, overlapping genes of significance were identified in a meta-analysis using the Fisher’s procedure. Finally, we performed gene expression analyses to confirm transcriptional changes. We identified 24 shared genes with Bonferroni correction (*P*_combined_ < 2.31E-04), and five (*FOXP1*, *CAMK2G*, *CLEC2D*, *LBH*, and *SLC2A4RG*) had significant expression differences in MS and IS gene expression omnibus data sets. These meaningful shared genes between IS and MS shed light on the underlying genetic etiologies shared by the diseases. Our results provide a basis for in-depth genomic studies of associations between MS and IS.

## Introduction

Multiple sclerosis and IS are two major neurological diseases with serious sequelae such as motor and/or sensory disabilities, cognitive impairment, and mental disorders (Compston and Coles, 2008; Johnson et al., 2016). Researchers are increasingly interested in the genetics of complex human diseases associated with high personal, family, and social burden, including MS and IS. Clarifying the genetics of these conditions can create a “strategic hub” for further studies to prioritize targeted treatments.

Multiple sclerosis is a chronic autoimmune disease in the CNS mainly characterized by demyelination in brain and spinal cord (Compston and Coles, 2008). The etiology of MS is heterogeneous, and both the environment and genetics are influential components (Dendrou et al., 2015). As environmental variants are complicated, the genetic variants are regarded as a possible direction to make progress in immune-related mechanisms and therapies (Belbasis et al., 2015; Dendrou et al., 2015). Numerous large-scale GWASs revealed more than 200 susceptible loci of MS, such as *HLA*, forkhead box protein P1 (*FOXP1*), *IQCB1*, *SLC9A9*, and *CD226* (Liu et al., 2017a; International Multiple Sclerosis Genetics Consortium, 2019). The pathway-based analysis results of MS risk variants were mostly enriched in immune- and/or inflammation-related processes (International Multiple Sclerosis Genetics Consortium, 2013; Liu et al., 2017b, c). Decades of progress in genetics have shed light on MS mechanisms and therapies. Several US Food and Drug Administration (FDA)-approved drugs have been used in patients with MS, such as ocrelizumab, daclizumab, and mitoxantrone (Baecher-Allan et al., 2018).

Ischemic stroke accounts for 70% to 85% of stroke cases, which is the most common cause of death and long-term disability worldwide (Khan et al., 2013; Johnson et al., 2016). Many pharmacologic therapies have been used to reduce damage and improve the prognosis of IS in preclinical and clinical trials, including thrombolytic, antithrombotic, antioxidation, and neuroprotective agents (Gladstone et al., 2002; Yu et al., 2005; Chamorro et al., 2016; Wechsler et al., 2018). To date, intravenous alteplase is the primary FDA-approved drug for IS. However, thrombolysis benefits a limited number of patients with IS because of the limited “therapeutic time window” and the unpredictable outcomes of recanalization. More attention should be paid to develop adjuvant treatments for IS that target inflammation and oxidative stress (Chamorro et al., 2016). Recent GWASs have uncovered ∼35 genetic risk regions for IS and hemorrhagic stroke (Dichgans et al., 2019). Early GWASs of IS identified and replicated the significant relationship for IS at *ABO*, cardioembolic stroke near *PITX2* and *ZFHX3*, and for large-vessel stroke at *HDAC9* and the 9p21 locus (Bellenguez et al., 2012; Traylor et al., 2012; Malik et al., 2016). Malik et al. (2018) implemented a comprehensive multiancestry GWAS using ancestral meta-analyses of 67,162 IS cases and 454,450 healthy controls. Twenty-two new loci were discovered and used for further pathway and drug–target analyses (Malik et al., 2018). Torres-Aguila et al. (2019) found that 14q24.3 locus was associated with leukocyte counts during the first 24 h after IS. Genetic and related functional studies are considered one of the breakthrough points for IS therapy (Dichgans et al., 2019). So far, because of the limitation of known GWAS data, the common gene variant that had been proved both MS and IS risk gene was *SLC44A2* (*P-*_*MS*_ = 3.5E-09, *P-*_*IS*_ = 4.72E-08) (International Multiple Sclerosis Genetics Consortium et al., 2013; Malik et al., 2018).

Clinical studies revealed an increased prevalence of IS in MS patients compared with controls (Marrie et al., 2015; Hong et al., 2019). Tseng et al. (2015) enrolled 1,174 Chinese MS patients and 4,696 matched healthy Chinese controls. After following up for 5 years, they found that the MS group got a higher risk of stroke [hazard ratio (HR) = 12.1 for the first year; HR = 4.69 for the rest years] than the control cohort. As more immunological characteristics of human complex diseases such as MS have been uncovered, the neuroinflammatory mechanisms of IS should have deeper exploration (Fu et al., 2015). For example, the natural killer (NK) cells aggravated the infarct area after IS, but their influence on MS was proven to be dependent on the course of disease (Chanvillard et al., 2013; Gan et al., 2014). Based on preclinical studies, more immunomodulatory drugs have been administered in IS clinical translation experiments including natalizumab (Elkins et al., 2017) and fingolimod (Zhu et al., 2015; Tian et al., 2018), which are the FDA-approved agents for MS.

Here, we focus on the commonality genetic etiologies between MS and IS. First, we conducted gene-based testing of MS and IS GWAS data sets. Next, a meta-analysis was conducted to sort the shared significant genes out. Finally, we implemented differential expression analyses for shared significant genes via four gene expression omnibus (GEO) data sets.

## Materials and Methods

### GWAS Data Set

The large-scale MS GWAS data set consisted of 9,772 MS cases from the IMSGC and 17,376 controls from the WTCCC2. And cases were gathered by 23 teams operating in 15 countries. All individuals were self-reported as being of European descent. The original research conducted sample and single-nucleotide polymorphism (SNP) QC to create SNP metric strainers. In sample QC, Bayesian clustering and principal components analysis were mainly used to identify qualified samples. Researchers developed and utilized two novel methods (automated cluster checking and beta-binomial model) in SNP QC to exclude SNPs identified because of incorrect genotype calling. Finally, 464,357 autosomal SNPs from the entire data set were obtained for further analysis (International Multiple Sclerosis Genetics Consortium et al., 2011).

The IS data set was summarized from the METASTROKE collaboration’s discovery stage. In this stage, genotyped data from 12 case–control GWASs correspond to 10,307 IS cases and 19,326 controls (ASGC: 1,162 IS cases and 1,244 controls; BRAINS: 371 IS cases and 2,640 controls; GASROS_affy: 485 IS cases and 3,030 controls; GASROS_illumina: 296 IS cases and 377 controls; GEOS: 448 IS cases and 498 controls; HPS: 588 IS cases and 571 controls; ISGS-SWISS: 1,014 IS cases and 1,370 controls; MILANO: 366 IS cases and 407 controls; VISP: 1,723 IS cases and 1,047 controls; WHI: 306 IS cases and 2,170 controls; WTCCC2-D: 1,174 IS cases and 797 controls; WTCCC2-UK: 2,374 IS cases and 5,175 controls; all individuals in the discovery stage were Caucasian). After QC by logistic regression analysis and meta-analysis, 9,541,572 SNPs were available for gene-based testing (Malik et al., 2016).

### Gene-Based Test of MS and IS GWAS Using VEGAS

We utilized the VEGAS2 to execute more elastic gene-based testing for MS and IS GWAS. The chief distinguishing feature of VEGAS2 is that this approach is computationally feasible and can be applied to any GWAS experimental design by using the summary GWAS data (Mishra and Macgregor, 2015). After uploading the SNP information (rs-number and *P* value of both GWASs) to VEGAS2, we selected 1000G EUROPEAN as the population reference group. For considering both the physical position and LD profiles of SNPs with others in gene-based testing, we chose “0kbldbin outside gene and SNPs in LD *r*^2^ > 0.8” (Christoforou et al., 2012). For each gene definition, *P* values of SNPs were first changed to upper tail χ^2^ statistics with one degree of freedom (*df*) in the gene definition. And then, if SNPs are in linkage equilibrium, the statistics of gene-based tests would have a χ^2^ distribution with *n df* under the null hypothesis. We used the reference population group of 1000 Genomes European and the model of Σ (*n* × *n* matrix of LD [*r*] values) for SNP correlation, because LD for the n SNPs occurs frequently. Significance was tested with reference to the aggregate χ^2^ statistics for each gene to simulated repetition from a multivariate normal distribution in which mean = 0 and variance = Σ. The empirical *P* value of every gene was calculated via the formula, *P* = *r* + 1/m + 1, where *r* was the number of cases where the simulation statistics surpassed the observed statistics, and *m* was the number of simulations (Mishra and Macgregor, 2015). In this gene-based testing, we screened the shared genes with nominal significance respectively (*P-*_*MS*_ < 0.05, *P-*_*IS*_ < 0.05).

### Meta-Analysis of MS and IS GWAS

To combine *P* values calculated by VEGAS2 for MS and IS GWAS, we used Fisher’s method to perform meta-analysis for every overlapped gene. For a given gene, the formula for the statistic is as follows:

$${\text{x}}^{2}=-2\,\sum_{i=1}^{k}\ln\left(P_{i}\right)$$

For the *i*_*th*_ study, *P*_*i*_ is the *P* value of the genes, and *k* is the entire count of studies. *x*^2^ abides by a *c*^2^ distribution with 2k degrees of freedom (Begum et al., 2012). The gene-based meta-analysis was performed with R software.^1^

Following the meta-analysis, we performed Bonferroni correction to control type I error. The combined *P* value was less than 0.05/2*n*, where *n* was the number of shared genes with previous nominal *P* value (*P* < 0.05).

### MS and IS Case–Control Gene Expression Analysis

We further investigated the differential expression of shared genes in the MS and IS patients and healthy control subjects using gene expression data sets from the NCBI GEO database.^2^

Immune cells from whole blood are the typical samples for gene expression analysis in patients with MS. PBMCs from 12 MS female patients under Poser’s criteria and 15 unrelated female controls were isolated from whole blood. The extracted samples were tested on the Affymetrix Gene Chip Human Genome U133 Plus 2.0 Array (Kemppinen et al., 2011). The expression data were obtained from GEO series GSE21942. In GEO series GSE43591, samples were extracted from 10 relapsing–remitting MS patients diagnosed in accordance with the criteria of McDonald et al. (2001) and 10 age- and sex-matched controls. T cells were purified through CD14^+^ removal sorting and CD3^+^-positive selection from PBMCs. Transcriptional data were examined with Human Genome HG-U133 plus 2.0 arrays (Jernas et al., 2013).

Ischemic stroke case–control expression analyses were performed to identify differentially expressed genes in the GEO series. GEO series GSE16561 provided the raw mRNA expression data from peripheral whole blood of a study of 39 IS patients and 24 healthy controls (O’Connell et al., 2017). More information about the inclusion and exclusion criteria was provided in the original study (O’Connell et al., 2017). RNA data from the blood of 23 control samples and 69 cardioembolic stroke samples were analyzed in GEO series GSE58294. Cardioembolic stroke is a subtype of IS and has unique genetic traits (Stamova et al., 2014). In the original research, 69 cardioembolic stroke samples were collected from 23 patients at three time points to monitor transcriptional changes in the first 24 h. Twenty-three subjects without symptomatic vascular disease history were recruited as VRFCs (Stamova et al., 2014). And we used all the 69 cardioembolic stroke samples as a whole (including the samples of three time points) to compare with the samples from VRFCs.

To calculate differential expression between case–control samples, we utilized GEO2R and performed GEO query and limma R packages (Barrett et al., 2013). Among four differential expression data sets, those with the smallest *P* value for each shared gene in MS and IS were selected for further analysis.

## Results

### Gene-Based Test for MS and IS With VEGAS2

We uploaded the 464,357 MS and 9,541,572 IS SNP rs IDs and associated *P* values for gene-based analysis. We sorted 1,353 genes with *P*-_*MS*_ < 0.05 from a total of 14,811 MS gene sets obtained by VEGAS2. After Bonferroni correction (*P* < 0.05/14,811 = 3.38E-06), 47 non-major histocompatibility complex (MHC) and MHC risk variants were sorted including known risk genes such as *AHI1*, *RGS1*, *SP140*, *IQCB*, *IL2RA*, *TNFRSF1A*, *CLEC16A*, *GALC*, *TNFSF14*, and *CYP24A1*. The good applicability of VEGAS2 for gene-based test was proven by the well-replicated genes above. Information of the gene-wide significant genes of MS is shown in Supplementary Table S1. In IS data sets, 1,290 genes with *P*-_*IS*_ < 0.05 were screened out from 21,913 IS genes via VEGAS2. However, no IS variant achieved the strict threshold (*P-*_*IS*_ < 0.05/21,913 = 2.28E-06). And we utilized a looser significance threshold (*P* < 1.00E-04) and identified two genes that satisfied this threshold set: *ZYX* (*P* < 2.60E-05) and *NCR3LG1* (*P* < 8.10E-05).

After making an intersection between 1,353 MS genes and 1,290 IS genes (*P*-_*MS*_ < 0.05, *P-*_*IS*_ < 0.05), we obtained 108 nominal shared genes. We performed meta-analysis for the 108 shared genes and obtained the *P*_–combined_ for each gene. And then 24 overlapped significant genes were identified with *P*_–combined_ < 2.31E-04 (0.05/2^∗^108), including *LBH* at 2p23.1, *STAT4* at 2q32.2-q32.3, *FOXP1* at 3p13, *TEC* at 4p12-p11, *MICB*/*NFKBIL1* at 6p21.33, *IL6* at 7p15.3, *CAMK2G* at 10q22.2, *B4GALNT1* at 12q13.3, *BRAP* at 12q24.12, *CLEC2D*/*CLECL1* at 12p13.31, *HECTD4*/*NAA25* at 12q24.13, *OS9* at 12q13.3-q14.1, *NUDT14* at 14q32.33, *PDE4C* at 19p13.11, *SLC44A2* at 19p13.2, and *LIME1*/*RTEL1*/*RTEL1-TNFRSF6B*/*SLC2A4RG*/*ZBTB46*/*ZGPAT* at 20q13.33. The detailed results are listed in Table 1.

**TABLE 1:** The overlapped significant gene sets screened by VEGAS of multiple sclerosis (MS) and ischemic stroke (IS).

		MS				IS				
Chr	Gene	*P* value	nSNPs	Top-SNP	Top-SNP *P* value	*P* value	nSNPs	Top-SNP	Top-SNP *P* value	*P*_combined_
2	*LBH*	8.34E-04	8	rs17321999	1.41E-04	1.39E-03	105	rs79251390	2.45E-04	1.70E-05
2	*STAT4*	1.00E-04	20	rs6752770	2.48E-04	4.17E-02	239	rs35672585	1.25E-03	5.58E-05
3	*FOXP1*	3.53E-04	137	rs1499895	4.51E-05	3.80E-02	700	rs7616330	9.97E-05	1.64E-04
4	*TEC*	4.85E-04	37	rs17471024	6.21E-05	1.11E-02	494	rs73817607	2.69E-04	7.07E-05
6	*MICB*	1.00E-06	4	rs2844498	1.58E-54	4.27E-03	213	rs2844498	7.51E-04	8.66E-08
6	*NFKBIL1*	1.00E-06	3	rs6929796	1.05E-32	2.39E-02	68	rs114205738	6.99E-04	4.43E-07
7	*IL6*	3.60E-05	2	rs2066992	1.89E-05	3.58E-02	20	rs2069832	1.32E-02	1.88E-05
10	*CAMK2G*	4.52E-04	5	rs2675671	9.40E-04	2.44E-02	92	rs4746152	5.25E-03	1.37E-04
12	*B4GALNT1*	7.10E-05	2	rs10083154	3.45E-06	3.98E-02	27	rs715930	1.94E-02	3.89E-05
12	*BRAP*	1.93E-02	5	rs11065987	3.83E-03	3.96E-04	45	rs11065987	5.05E-05	9.77E-05
12	*CLEC2D*	1.87E-04	6	rs3764021	1.24E-05	6.40E-03	105	rs7968401	1.53E-04	1.75E-05
12	*CLECL1*	3.16E-04	5	rs10466829	1.09E-05	3.26E-04	106	rs2401391	1.55E-04	1.76E-06
12	*HECTD4*	3.22E-02	11	rs11066188	2.14E-03	3.16E-04	255	rs10850034	2.49E-05	1.27E-04
12	*NAA25*	1.34E-02	7	rs17696736	1.93E-03	1.21E-04	105	rs17696736	1.13E-05	2.32E-05
12	*OS9*	9.80E-05	2	rs799265	7.96E-06	1.38E-02	50	rs76809208	3.47E-03	1.96E-05
14	*NUDT14*	2.29E-02	2	rs11625862	1.60E-02	3.33E-04	26	rs11625865	9.99E-05	9.75E-05
19	*PDE4C*	4.54E-04	7	rs4808762	1.06E-06	1.78E-02	101	rs11667487	1.65E-03	1.03E-04
19	*SLC44A2*	3.16E-04	4	rs8106664	1.78E-04	5.42E-03	142	rs7250421	5.95E-04	2.45E-05
20	*LIME1*	8.20E-05	2	rs2427536	6.78E-05	8.91E-03	19	rs1056441	1.64E-03	1.11E-05
20	*RTEL1*	1.76E-04	6	rs6011002	2.36E-04	6.07E-03	192	rs145832440	6.13E-04	1.58E-05
20	*RTEL1-TNFRSF6B*	1.47E-04	6	rs6011002	2.36E-04	5.58E-03	205	rs145832440	6.13E-04	1.23E-05
20	*SLC2A4RG*	8.60E-05	2	rs2427536	6.78E-05	1.02E-02	20	rs1056441	1.64E-03	1.31E-05
20	*ZBTB46*	3.04E-03	12	rs6062314	8.26E-07	5.42E-03	302	rs150589984	2.18E-04	1.98E-04
20	*ZGPAT*	1.46E-03	5	rs1151625	1.04E-04	5.93E-03	98	rs6011040	1.22E-03	1.10E-04

*Chr, chromosome; SNP, single-nucleotide polymorphism; nSNPs, number of SNPs; TopSNP, the most significant SNP in related gene.*

### Differential Expression Analyses of Overlapped Genes

We analyzed and integrated GEO profiles of MS and IS to investigate the differential expression levels of shared genes. Notably, 16 of 24 common genes were determined to have significant changes in at least one of the four GEO data sets with *P* < 2.08E-03 (0.05/24) (Table 2).

**TABLE 2:** The expression alteration of shared gene sets for multiple sclerosis and ischemic stroke.

	MS		IS	
Gene	GSE21942	GSE43591	GSE16561	GSE58294
*B4GALNT1*	8.53E-02	9.83E-02	3.48E-01	2.03E-02
*BRAP*	4.87E-01	1.44E-02	4.13E-01	1.71E-20
*CAMK2G*	4.15E-04	3.09E-06	5.81E-01	5.14E-07
*CLEC2D*	9.11E-04	7.91E-02	2.68E-07	1.78E-01
*CLECL1*	7.42E-01	4.19E-01	2.94E-01	6.30E-06
*FOXP1*	2.35E-09	1.17E-02	4.27E-02	1.55E-05
*HECTD4*	7.59E-02	1.34E-02	—	8.83E-05
*IL6*	1.27E-02	4.99E-01	9.05E-01	1.02E-01
*LBH*	3.52E-02	5.60E-04	5.45E-06	7.92E-01
*LIME1*	3.35E-02	9.94E-03	2.33E-07	1.16E-09
*MICB*	6.07E-01	2.05E-01	8.59E-01	2.45E-04
*NAA25*	2.09E-01	5.01E-02	9.57E-01	3.92E-08
*NFKBIL1*	3.63E-01	6.31E-01	2.21E-01	3.33E-06
*NUDT14*	1.51E-04	8.09E-01	9.00E-03	2.53E-02
*OS9*	2.99E-01	8.58E-01	1.41E-04	1.24E-12
*PDE4C*	2.52E-02	2.01E-01	2.40E-01	8.74E-02
*RTEL1*	1.43E-01	1.73E-01	2.53E-01	8.01E-01
*RTEL1-TNFRSF6B*	2.20E-03	9.24E-03	—	5.42E-01
*SLC2A4RG*	7.21E-02	1.29E-03	5.07E-05	3.18E-01
*SLC44A2*	4.03E-02	9.87E-02	4.73E-04	1.27E-03
*STAT4*	1.47E-01	7.80E-01	3.93E-03	9.05E-01
*TEC*	7.27E-01	5.51E-01	4.71E-01	9.03E-02
*ZBTB46*	5.81E-01	7.17E-01	7.52E-01	1.06E-02
*ZGPAT*	5.70E-02	6.93E-04	6.20E-03	8.85E-03

*GEO series, GSE21942, GSE43591, GSE16561, and GSE58294. “—”, data from the related GEO database are not available.*

In MS expression profiles, the transcriptional levels of *CAMK2G* (*P* = 4.15E-04), *CLEC2D* (*P* = 9.11E-04), *FOXP1* (*P* = 2.35E-09), and *NUDT14* (*P* = 1.51E-04) in the GEO data set GSE21942 (Supplementary Table S2) and *CAMK2G* (*P* = 3.09E-06), *LBH* (*P* = 5.60E-04), *SLC2A4RG* (*P* = 1.29E-03), and *ZGPAT* (*P* = 6.93E-04) in MS patients were markedly changed compared to controls (Supplementary Table S3). In samples from peripheral whole blood, *CLEC2D* (*P* = 2.68E-07), *LBH* (*P* = 5.45E-06), *LIME1* (*P* = 2.33E-07), *OS9* (*P* = 1.41E-04), *SLC2A4RG* (*P* = 5.07E-05), and *SLC44A2* (*P* = 4.73E-04) in the acute IS case–control set (GSE16561) (Supplementary Table S4) and *BRAP* (*P* = 1.71E-20), *CAMK2G* (*P* = 5.14E-07), *CLECL1* (*P* = 6.30E-06), *FOXP1* (*P* = 1.55E-05), *HECTD4* (*P* = 8.83E-05), *LIME1* (*P* = 1.16E-09), *MICB* (*P* = 2.45E-04), *NAA25* (*P* = 3.92E-08), *NFKBIL1* (*P* = 3.33E-06), *OS9* (*P* = 1.24E-12), and *SLC44A2* (*P* = 1.27E-03) in the cardioembolic stroke case–control set (GSE58294) (Supplementary Table S5) showed significantly different expression.

It was remarkable that there were five overlapped genes with a significant expressed difference in at least one data set of each disease GEO data sets (*P* < 2.08E-03) (Table 3). The expression difference of every individual gene presented the same tendency between two GEO data sets in MS, and *CAMK2G* was also upregulated in IS GEO data sets, whereas three of five genes (*CLEC2D*, *LBH*, and *SLC2A4RG*) had opposite alterations in GSE16561 and GSE58294, which might be due to the heterogeneity of IS and its subtypes. *FOXP1* expression in patients was remarkably increased in every data set compared to controls [GSE21942: log fold change (FC) = 1.21, GSE43591: logFC = 0.528, GSE16561: logFC = 0.15, and GSE58294: logFC = 0.34].

**TABLE 3:** Significant shared gene sets of multiple sclerosis and ischemic stroke in GEO data sets.

	Multiple sclerosis and control				Ischemic stroke and control			
Gene	GSE21942		GSE43591		GSE16561		GSE58294	
	*P* value	logFC	*P* value	logFC	*P* value	logFC	*P* value	logFC
*CAMK2G*	**4.15E-04**	–0.551	**3.09E-06**	–0.44	5.81E-01	0.043117	**5.14E-07**	0.352815
*CLEC2D*	**9.11E-04**	0.489	7.91E-02	0.24	**2.68E-07**	–0.66082	1.78E-01	0.180726
*FOXP1*	**2.35E-09**	1.21	1.17E-02	0.528	4.27E-02	0.149791	**1.55E-05**	0.339692
*LBH*	3.52E-02	–0.321	**5.60E-04**	–0.553	**5.45E-06**	–0.55397	7.92E-01	0.02761
*SLC2A4RG*	7.21E-02	–0.205	**1.29E-03**	–0.332	**5.07E-05**	–0.24643	3.18E-01	0.07236

*logFC, log2-fold change. Bolded *P* values of genes achieved Bonferroni-corrected significance, adjusted for the number of shared genes present in each expression data set.*

## Discussion

With the development of high-throughput technology, more risk loci of MS and IS have been gradually identified (International Multiple Sclerosis Genetics Consortium et al., 2013; Moutsianas et al., 2015; Malik et al., 2018). However, genes shared by the two diseases remain elusive. In this study, we implemented a three-part process to assess GWAS data sets. In Part 1, we conducted independent gene-based association analyses using two GWAS data sets and VEGAS2. In Part 2, we performed a Fisher’s meta-analysis of nominally significant common genes (*P-*_*MS*_ < 0.05, *P-*_*IS*_ < 0.05), and 24 shared genes satisfied the strict threshold *P* value (*P* < 2.31E-04). In Part 3, we listed expression information of shared genes in four GEO data sets and found that *FOXP1*, *CAMK2G*, *CLEC2D*, *LBH*, and *SLC2A4RG* presented significant differential expression in both MS and IS case–control profiles.

### *FOXP1* Located at 3p13

Forkhead box protein P1 protein is a member of the forkhead box (FOX) family. The forkhead TF plays vital roles in diverse cell and tissue processes including development, aging, metabolism, and cancer (Coffer and Burgering, 2004; Wijchers et al., 2006). *FOXP1* is also critical in the development and function of immune cells. In peritonitis models, Shi et al. (2008) illustrated that *FOXP1* upregulation blunted monocyte development and macrophage biological activities by inhibiting the production of *c-Fms*/macrophage colony-stimulating factor receptor. Via Smad2/Smad3 and transforming growth factor β (TGF-β) signaling, increased FOXP1 expression restrained CD8^+^ T cells from proliferation and activation in cancer (Stephen et al., 2014). Combined with *FOXP3*, *FOXP1* was demonstrated to play an essential role in maintaining expression of CD25, SATB1, and CTLA-4 and responsiveness to interleukin 2 in regulatory T cells (Konopacki et al., 2019). *FOXP1* was also reported as a crucial negative transcriptional modulator in the differentiation of CD4^+^ follicular helper T cells (Wang et al., 2014). Similarly, *FOXP1* is a fundamental TF in the early development of B cells (Hu et al., 2006), and abnormal *FOXP1* upregulation leads to a reduction and irregular distribution of B cells in germinal centers (Sagardoy et al., 2013). *FOXP1* is associated with both CNS development and abnormalities. Braccioli et al. (2017) reported that *FOXP1* can adjust neural stem cells (NSCs) neurogenesis via Notch signaling and foster the differentiation of embryonic NSCs to neurons and astrocytes *in vitro*. Li et al. (2015) demonstrated that *FOXP1* has vital influences on modulating neuronal migration and morphogenesis in cortical regions. *FOXP1* mutations reportedly contribute to nervous system disorders including Huntington disease (Tang et al., 2012), autism (O’Roak et al., 2011; Chien et al., 2013), and epilepsy (Jay et al., 2019).

In this study, *FOXP1* exhibited an elevated tendency of expression in both MS and IS patients compared to controls (GSE21942: logFC = 1.21; GSE43591: logFC = 0.528; GSE16561: logFC = 0.15, and GSE58294: logFC = 0.34). FOXP1 was significantly more than twofold upregulated in the PBMC of MS (*P* = 2.35E-09, logFC = 1.21) and significantly 1.3-fold upregulated in the blood cells of cardioembolic stroke (*p* = 1.55E-05, logFC = 0.34). The IMSGC assessed two sets of GWAS data and identified rs9828629/FOXP1 (*P* = 1.9E-10, odds ratio [OR] = 1.08) as an immune-related risk variant linked to MS (International Multiple Sclerosis Genetics Consortium et al., 2013). In research on atherosclerosis, Bot et al. (2011) illustrated that FOXP1 was expressed in diverse cell types and was related with stable plaques through the TGF-β pathway. As atherosclerosis is one cause of large vessel disease, a major subtype of IS, there may be more associations between FOXP1 and IS. In summary, *FOXP1* could be a potential target in terms of its multiple functions in the immune and nervous systems.

### *CAMK2G* Located at 10q22.2

*CAMK2G* encodes CaMKIIγ, an isoform of calcium (Ca^2+^)/calmodulin-dependent protein kinase II (CaMKII) that participates in Ca^2+^-related biological activities (Hudmon et al., 2001; Rokita and Anderson, 2012). In a model of acute ischemia/reperfusion, *CAMK2G*/CaMKIIγ improved neuronal survival by activating nuclear factor κB signaling (Ye et al., 2019). *CAMK2G* was identified as an enhancer gene for coronary artery disease in a GWAS meta-analysis (Gong et al., 2018). CAMKIIγ was also a key modulator to repress macrophage phagocytosis and enhance the necrosis of atherosclerotic plaques (Doran et al., 2017). We found that *CAMK2G* (*P* = 5.14E-07) was 1.3-fold upregulated in cardioembolic stroke compared to controls. In addition, *CAMK2G* could act through classical inflammatory pathways such as mTORC1 and STAT3, both of which play vital roles in MS (Ma et al., 2017; Meng et al., 2017).

### *CLEC2D* Located at 12p13.31

*CLEC2D* encodes lectin-like transcript 1 (LLT1) and is located next to the NK gene complex (Yokoyama and Plougastel, 2003; Germain et al., 2010). LLT1 was identified as a negative ligand for NKR-P1A (CD161) receptor in humans (Rosen et al., 2005). The interaction of CD161 on NK cells with matched LLT1 can reportedly repress NK cell-mediated cytotoxicity (Aldemir et al., 2005). Llibre et al. (2016) illustrated that LLT1/CD161 interaction influences B-cell development in human germinal centers. Toll-like receptor (TLR)-activated dendritic cells and TLR or B-cell receptor-activated B cells express LLT1 (Rosen et al., 2008). LLT1 is also expressed by monocyte/macrophage in RA joints, and soluble LLT1 is elevated in patient serum (Chalan et al., 2015). Here, we detected that the expression of *CLEC2D* (*P* = 9.11E-04, logFC = 0.49) was significantly altered in PBMCs from MS patients. However, the expression of *CLEC2D* in IS cases was downregulated by 0.62-fold compared to controls. The distinct alterations of *CLEC2D* expression in MS and IS need further study.

### *SLC2A4RG* Located at 20q13.33

SLC2A4 regulator encoded by *SLC2A4RG* was identified as TF binding to the SLC2A4 promoter district where it regulates expression (Oshel et al., 2000; Knight et al., 2003). In a study of Huntington disease, *SLC2A4RG* was found to cooperate with a key *cis*-element and shuttle to and from the nucleus (Tanaka et al., 2004). A recent large GWAS demonstrated that rs2256814/*SLC2A4RG* (*P* = 3.5E-9, OR = 1.08) was a novel MS susceptibility gene with immune function (International Multiple Sclerosis Genetics Consortium et al., 2013). Furthermore, Dhaouadi et al. (2014) stated that SLC2A4RG could slightly induce TGFB1 expression in atherosclerosis. Notably, SLC2A4RG showed differential expression in MS and IS GEO data sets (GSE43591, *P* = 1.29E-03; GSE16561, *P* = 5.07E-05).

### *LBH* Located at 2p23.1

Limb-bud and heart (*LBH*) encodes a transcription modulator that regulates cell development in multiple tissues (Ai et al., 2008; Al-Ali et al., 2010; Ekwall et al., 2015). *LBH* and related variants are reportedly associated with autoimmune diseases such as SLE, RA, and celiac disease (Zhernakova et al., 2011; Yu et al., 2013; Chang et al., 2016). Wnt signaling regulates the expression of *LBH* in tumors and the epithelium (Rieger et al., 2010). In the CNS, the Wnt pathway is related with male-specific genes of IS and positively impacts the regenerative process after injury (Tian et al., 2012; Lambert et al., 2016). Researchers integrated Alzheimer’s disease-GWAS data and identified *LBH* as a pathogenic gene of amyloid β accumulation that is tightly linked to immune system (Mhatre et al., 2015; Yamaguchi-Kabata et al., 2018). In our study, *LBH* was significantly downregulated both in T cells from PBMCs of patients with MS (*P* = 5.60E-04, logFC = −0.55) and blood cells from patients with IS (*P* = 5.45E-06, logFC = −0.55). The potential immunomodulatory function of *LBH* can assist us in uncovering common mechanisms between MS and IS.

Although we conducted powerful gene-based tests that are effective extensions of traditional GWASs to discover shared genes, our study has several limitations. First, subthreshold variants will undermine the significance of the causal SNPs in the same gene region. Next, IS consists of several subtypes with genetic heterogeneity. More refined data of IS subtypes should be excavated for further analyses. Furthermore, the single sample type and limited case numbers are two major disadvantages of our GEO data. All GEO sample sources are from peripheral blood cells because they are safe and accessible in clinical settings. If possible, we will integrate brain-derived data that can directly reflect the differential expression of genes in neurological diseases.

## Conclusion

In summary, we performed flexible gene-based analysis to discover significant shared genes between MS and IS and analyzed their differential expression. These genes mainly participate in cell development and immune response, and both are associated with MS and IS. Our research reveals shared genetic etiologies between MS and IS and indicates new directions for future studies examining mechanisms and new therapeutic options.

## Data Availability Statement

All datasets analyzed for this study are included in the article and the Supplementary Files.

## Author Contributions

ZT and ZW conceived and designed the study for MS and IS. ZT administered the analyses and wrote the manuscript. YS and ZG was responsible for manuscript revision. YY and JG provided analyses support. All authors contributed to the article and approved the submitted version.

## Conflict of Interest

The authors declare that the research was conducted in the absence of any commercial or financial relationships that could be construed as a potential conflict of interest.

## Footnotes

1 http://www.r-project.org/

2 www.ncbi.nlm.nih.gov/geo

## Supplementary Material

TABLE S1 MS-associated genes with gene-wide significance.

TABLE S2 The expression data of shares gene sets from peripheral blood mononuclear cells in multiple sclerosis patients and controls (Series GSE21942).

TABLE S3 The expression data of shares gene sets from peripheral blood T-cells in multiple sclerosis patients and controls (Series GSE43591).

TABLE S4 The expression data of shares gene sets from whole blood RNA in ischemic stroke patients and controls (Series GSE16561).

TABLE S5 The expression data of shares gene sets from whole blood RNA in cardioembolic stroke patients and controls (Series GSE58294).

## Abbreviations

CNS: central nervous system
GEO: gene expression omnibus
GWASs: large-scale genome-wide association studies
IMSGC: International MS Genetics Consortium
IS: ischemic stroke
LD: linkage disequilibrium
MS: multiple sclerosis
PBMCs: peripheral blood mononuclear cells
QC: quality control
RA: rheumatoid arthritis
SLE: systemic lupus erythematosus
SNP: single nucleotide polymorphism
TF: transcription factor
VEGAS2: Versatile Gene-based Association Study-2 version 2
VRFCs: vascular risk factor controls
WTCCC2: Wellcome Trust Case Control Consortium 2.
